# Crystal structure and Hirshfeld surface analysis of 2-amino­pyridinium hydrogen phthalate

**DOI:** 10.1107/S2056989019012957

**Published:** 2019-10-08

**Authors:** V. Siva, M. Suresh, S. Athimoolam, S. Asath Bahadur

**Affiliations:** a Department of Physics, School of Advanced Sciences, Kalasalingam Academy of Research and Education, Krishnankoil - 626 126, India; b Condensed Matter Physics Laboratory, International Research Centre, Kalasalingam Academy of Research and Education, Krishnankoil - 626 126, India; c Department of Physics, Er. Perumal Manimekalai College of Engineering, Hosur 635 117, India; d Department of Physics, University College of Engineering, Anna University, Nagercoil 629 004, India

**Keywords:** crystal structure, organic salt, hetero-synthon, NLO, hydrogen bonding, Hirshfeld surface analysis

## Abstract

Amino­pyridine and phthalic acid are well known synthons for supra­molecular architectures for the synthesis of new materials for optical applications. The 2-amino­pyridinium hydrogen phthalate title salt, C_5_H_7_N_2_^+^·C_8_H_5_O_4_^−^, crystallizes in the non-centrosymmetric space group *P*2_1_. The nitro­gen atom of the –NH_2_ group in the cation deviates from the fitted pyridine plane by 0.035 (7) Å. The plane of the pyridinium ring and phenyl ring of the anion are oriented at an angle of 80.5 (3)° to each other in the asymmetric unit. The anion features a strong intra­molecular O—H⋯O hydrogen bond, forming a self-associated *S*(7) ring motif. The crystal packing is dominated by inter­molecular N—H⋯O hydrogen bonds leading to the formation of 2_1_ helices, with a *C*(11) chain motif. They propagate along the *b* axis and enclose *R*_2_^2^(8) ring motifs. The helices are linked by C—H⋯O hydrogen bonds, forming layers parallel to the *ab* plane. Hirshfeld surface analysis and two-dimensional fingerprint plots were used to investigate and qu­antify the inter­molecular inter­actions in the crystal.

## Abstract 2

New proton-transfer single crystals of 2-amino­pyridinium phthalate were obtained with the aim of synthesizing a new non-linear optical (NLO) material, which is achieved with the help of extensive hydrogen-bonding inter­actions between the ions.

## Chemical context   

Crystal engineering and the design of supra­molecular architectures are of significant inter­est owing to the technological applications of the resulting materials in the electronics and optical industries. Supra­molecular inter­actions such as charge-assisted hydrogen bonds and π–π inter­actions play an important role in crystal engineering as they lead to directional mol­ecular recognition events between mol­ecules or ions, and therefore mediate self-assembly of well-defined supra­molecular networks (Guelmami *et al.*, 2007 ▸; Prakash *et al.*, 2018 ▸; Siva *et al.*, 2017 ▸). Amine-based materials are part­ic­ularly important as they are synthesized by the condensation of the corresponding aldehydes and amines and exhibit strong inter­molecular hydrogen bonds between the electronegative acceptor and the N atom of the imine moiety. Pyridinium families are now considered to be potential materials for optical applications because of their flexibility in mol­ecular design, strength and thermal stability, which are derived from delocalized clouds of electrons. Another electronic field of research related to 2-amino­pyridinium salts is focused on their optical limiting and frequency-conversion applications (Liu *et al.*, 2015 ▸; Siva *et al.*, 2019 ▸). The present work is a part of a structural study of new proton-transfer compounds of 2-amino­pyridine with phthalic acid and the corresponding hydrogen-bonding inter­actions. The hydrogen bonding present in the crystal of the title salt was substanti­ated by Hirshfeld surface analysis.
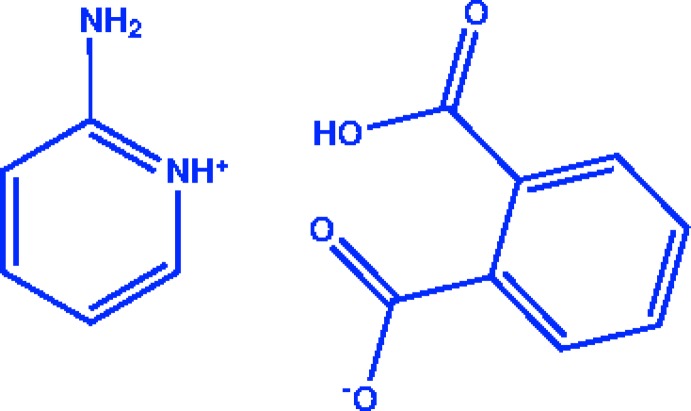


## Structural commentary   

**Figure 1:**
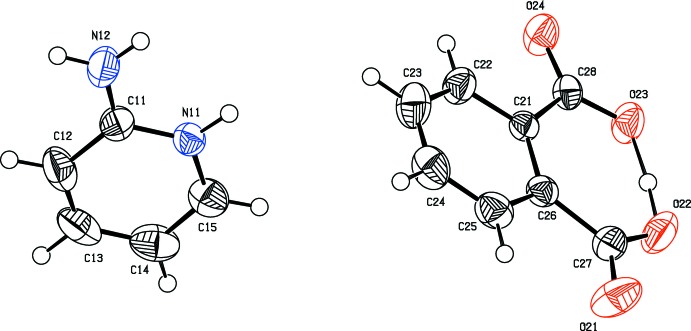
The asymmetric unit of the title salt, with atom labelling and 50% probability displacement ellipsoids.

The mol­ecular structure of the title salt is shown in Fig. 1 ▸. Protonation on the N-atom site of the pyridine ring, atom N11, is confirmed by the elongated C—N bond distances [C11—N11 = 1.341 (8) Å and C15—N11 = 1.357 (9) Å] and the enlarged C11—N11—C15 bond angle of 122.3 (6)°. The nitro­gen atom of the –NH_2_ group in the cation deviates from the pyridine ring plane (r.m.s. deviation = 0.0062 Å) by 0.035 (7) Å. The planes of the pyridinium ring of the cation and the phenyl ring of the anion are oriented at a dihedral angle of 80.5 (3)° in the asymmetric unit. In the anion the twisting of the carboxyl planes out of the benzene ring is negligible [planes O21/O22/C27 and O23/O24/C28 are inclined to the benzene ring (C21–C26) by 1.3 (8) and 0.7 (7)°, respectively], because of the strong O22—H22*A*⋯O23 intra­molecular hydrogen bond (Fig. 1 ▸ and Table 1 ▸), which makes a self-associated *S*(7) ring motif.

## Supra­molecular features   

2-Amino­pyridine and phthalic acid are known materials for structure-extension properties, which connect the mol­ecules in the supra­molecular assembly. These supra­molecular synthons are crystallized together not only to study the mol­ecular structure but also the crystal packing *via* inter­molecular inter­actions. This structure-extension property of the synthon mol­ecules is generally exploited for possible non-centrosymmetric materials, which are desired as they possess many applications. The structure extension of the mol­ecules is possible by linear (chain *C* motifs) and cyclic (ring *R* motifs) hydrogen-bonding associations. This was accomplished in the title compound, which exhibits non-linear optical (NLO) properties, because of the extensive inter­molecular inter­actions.

**Figure 2:**
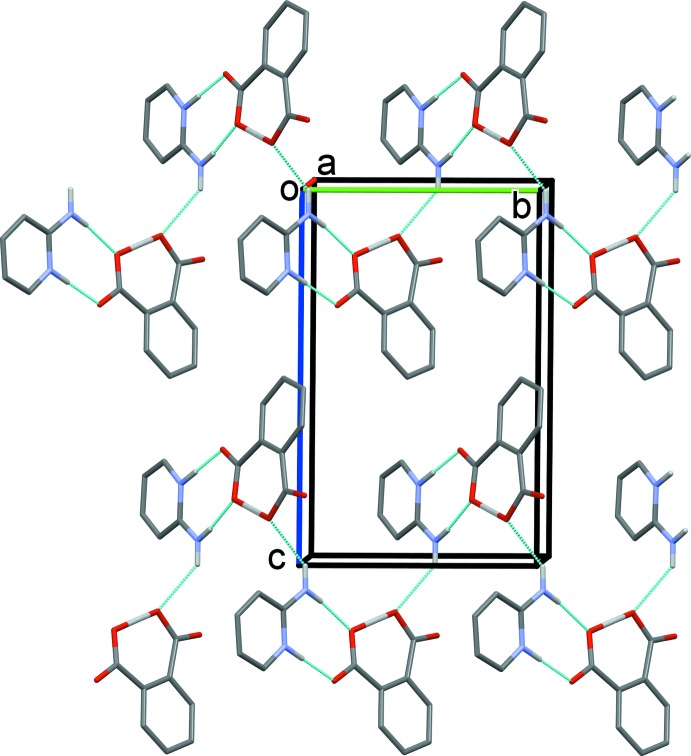
The crystal packing of the title salt viewed along the *a* axis. Hydrogen bonds are shown as dashed lines (Table 1 ▸). For clarity, the C-bound H atoms have been omitted.

**Figure 3:**
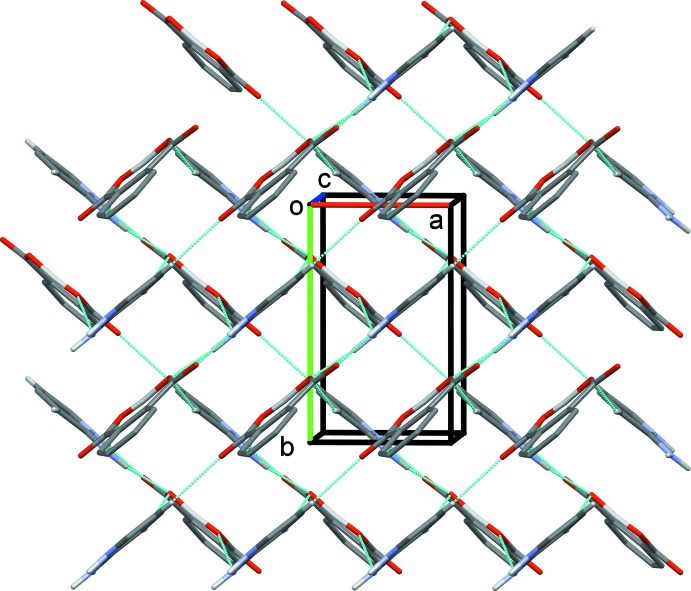
Packing of the title salt viewed along the *c* axis. Hydrogen bonds are shown as dashed lines (Table 1 ▸). For clarity, H atoms not involved in hydrogen bonding have been omitted.

The packing of the ions in the crystal is dominated by N—H⋯O and C—H⋯O hydrogen bonds (Table 1 ▸). A cation–anion hetero-synthon is formed *via* two N—H⋯O hydrogen bonds (N11—H1*N*⋯O24^i^ and N12—H12*A*⋯O23^i^), that enclose an 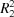
(8) ring motif (Fig. 2 ▸ and Table 1 ▸). These hetero-synthons are linked by a further N—H⋯O hydrogen bond (N12—H12*B*⋯O22^ii^), to form 2_1_ helices, with a *C*(11) chain motif, that propagate along the *b-*axis direction. The helices are linked by C—H⋯O hydrogen bonds, forming layers lying parallel to the *ab* plane (Fig. 3 ▸ and Table 1 ▸). There are no significant C—H⋯π or π–π contacts present in the crystal (*PLATON*; Spek, 2009 ▸).

## Hirshfeld surface analysis   

The Hirshfeld surface analysis (Spackman & Jayatilaka, 2009 ▸) and the associated two-dimensional fingerprint plots (McKinnon *et al.*, 2007 ▸) were performed with *CrystalExplorer17* (Turner *et al.*, 2017 ▸). The Hirshfeld surface is colour-mapped with the normalized contact distance, *d*_norm_, from red (distances shorter than the sum of the van der Waals radii) through white to blue (distances longer than the sum of the van der Waals radii).

**Figure 4:**
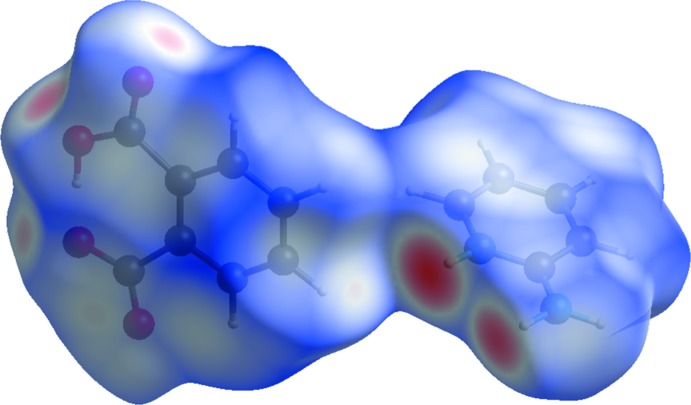
Hirshfeld surface for the title salt mapped over *d*_norm_, in the colour range −0.7098 to 1.1914 au.

The Hirshfeld surface (HS) of the title salt, mapped over *d*_norm_ in the colour range of −0.7098 to 1.1914 arbitrary units, is given in Fig. 4 ▸. The short inter­atomic contacts, *i.e*. the donors and acceptors of the hydrogen bonds (Table 1 ▸), are indicated by the red spots.

**Figure 5:**
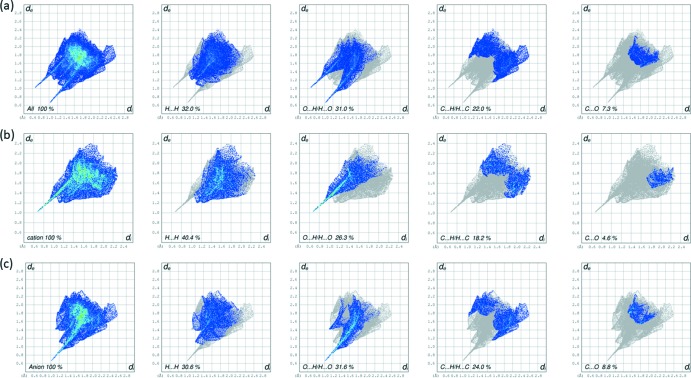
(*a*) The full two-dimensional fingerprint plot and the fingerprint plots delineated into H⋯H, O⋯H/H⋯O, C⋯H/H⋯C and C⋯O contacts for the title salt, (*b*) the full two-dimensional fingerprint plot and fingerprint plots delineated into H⋯H, O⋯H/H⋯O, C⋯H/H⋯C and C⋯O contacts for the cation, and (*c*) the full two-dimensional fingerprint plot and fingerprint plots delineated into H⋯H, O⋯H/H⋯O, C⋯H/H⋯C and C⋯O contacts for the anion (see Table 2 ▸ for further details).

The two-dimensional fingerprint plots for the title salt, the cation and the anion are given in Fig. 5 ▸. The relative percentage contributions of close contacts to the Hirshfeld surfaces of the title salt (Fig. 5 ▸ *a*), and the cation (Fig. 5 ▸ *b*) and anion (Fig. 5 ▸ *c*), are compared in Table 2 ▸.

For the title salt (Fig. 5 ▸ *a*), the most significant contributions to the HS are from H⋯H (32.0%), O⋯H/H⋯O (31.0%), C⋯H/H⋯C (22.0%) and C⋯O (7.3%) contacts. On examination of the contributions to the HS of the cation (Fig. 5 ▸ *b*) and anion (Fig. 5 ▸ *c*) individually, it can be seen that the cation makes the largest contribution to the H⋯H contacts (40.4%), while the anion makes the largest contributions to the O⋯H/H⋯O (31.6%), C⋯H/H⋯C(24.0%) and C⋯O (8.8%) contacts (see also Table 2 ▸).

## Database survey   

A search of the Cambridge Structural Database (Version 5.40, last update May 2019; Groom *et al.*, 2016 ▸) for 2-amino­pyridinium salts indicated that the crystal structures of more than 220 structures have been reported. A search for 2-amino­pyridinium benzoate salts gave 45 hits for 35 structures. The most significant in relation to the title salt are: 2-amino­pyridinium benzoate (LUPZOL; Odabaşoğlu *et al.*, 2003 ▸), 2-amino­pyridinium 2′-carb­oxy­biphenyl-4-carboxyl­ate (DEZCOC; Wang *et al.*, 2013 ▸), bis­(2-amino­pyridine) terephthalate (LAPMUL; Bis & Zaworotko), 2-amino­pyridinium isophthalate (Bis & Zaworotko, 2005 ▸) and bis­(2-amino­pyridinium) 2,5-di­carb­oxy­benzene-1,4-di­carboxyl­ate (Rodrigues *et al.*, 2012 ▸). In the crystals, the same hetero-synthon is formed *via* N—H⋯O hydrogen bonds. The CO_2_^−^ groups in general lie close to the plane of the benzene ring in LUPZOL, LAQGOA and ZARHOR; the dihedral angle varies from 1.85–6.09°. However, the corresponding dihedral angles in DEZCOC and LAPMUL are considerably lager; *ca* 47.92 and 23.97° in DEZCOC and 17.37° in LAPMUK. While DEZCOC crystallizes in a chiral space group, *P*3_2_, the other four compounds, LUPZOL, LAPMUL, LAQGOA and ZARHOR, crystallize in a centrosymmetric monoclinic space group (*P*2_1_/*c* or *P*2_1_/*n*) and hence do not exhibit NLO properties.

## Synthesis and crystallization   

A 1:1 mixture of 2-amino­pyridine and phthalic acid was heated to 313 K and stirred for 1 h before being poured into a petri dish and kept undisturbed for 25 days. Colourless block-shaped single crystals were obtained by the slow evaporation of a methanol and water (*v*:*v* = 20:80%) solution.

## Refinement   

Crystal data, data collection and structure refinement details are summarized in Table 3 ▸. The OH and NH H atoms were located in a difference-Fourier map and refined freely. The NH_2_ and C-bound H atoms were included in calculated positions and treated as riding atoms: N—H = 0.86 Å, C—H = 0.93 Å with *U*_iso_(H) = 1.2*U*_eq_(N, C).

## Supplementary Material

Crystal structure: contains datablock(s) I, Global. DOI: 10.1107/S2056989019012957/ff2163sup1.cif

Structure factors: contains datablock(s) I. DOI: 10.1107/S2056989019012957/ff2163Isup2.hkl

Supporting information file. DOI: 10.1107/S2056989019012957/ff2163Isup3.cml

CCDC reference: 1954730

Additional supporting information:  crystallographic information; 3D view; checkCIF report

## Figures and Tables

**Table 1:** Hydrogen-bond geometry (Å, °)

*D*—H⋯*A*	*D*—H	H⋯*A*	*D*⋯*A*	*D*—H⋯*A*
O22—H22*A*⋯O23	1.08 (10)	1.32 (10)	2.402 (7)	173 (9)
N11—H1*N*⋯O24^i^	0.93 (7)	1.78 (7)	2.705 (7)	180 (7)
N12—H12*A*⋯O23^i^	0.86	2.11	2.965 (7)	174
N12—H12*B*⋯O22^ii^	0.86	2.31	3.003 (8)	138
C12—H12⋯O23^iii^	0.93	2.53	3.374 (8)	151
C14—H14⋯O21^iv^	0.93	2.50	3.203 (9)	132

Symmetry codes: (i) 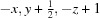; (ii) 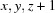; (iii) 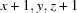; (iv) 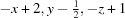.

**Table 2:** Relative percentage contributions of close contacts to the Hirshfeld surface for the title salt, the cation and the anion

Contact	cation+anion	cation	anion
H⋯H	32.0	40.4	30.6
O⋯H/H⋯O	31.0	26.3	31.6
C⋯H/H⋯C	22.0	18.2	24.0
C⋯O	7.3	4.6	8.8
N⋯H/H⋯N	2.5	5.1	0.4
C⋯C	2.5	2.2	2.6

**Table 3:** Experimental details

Crystal data	
Chemical formula	C_5_H_7_N_2_^+^·C_8_H_5_O_4_^−^
*M*_r_	260.25
Crystal system, space group	Monoclinic, *P*2_1_
Temperature (K)	293
*a*, *b*, *c* (Å)	5.1593 (6), 8.6124 (9), 13.5745 (19)
β (°)	97.087 (4)
*V* (Å^3^)	598.56 (13)
*Z*	2
Radiation type	Mo *K*α
μ (mm^−1^)	0.11
Crystal size (mm)	0.26 × 0.24 × 0.20

Data collection	
Diffractometer	Bruker SMART APEX CCD area-detector
No. of measured, independent and observed [*I* > 2σ(*I*)] reflections	6704, 2099, 1972
*R*_int_	0.023
(sin θ/λ)_max_ (Å^−1^)	0.595

Refinement	
*R*[*F* ^2^ > 2σ(*F* ^2^)], *wR*(*F* ^2^), *S*	0.059, 0.186, 1.24
No. of reflections	2099
No. of parameters	180
No. of restraints	1
H-atom treatment	H atoms treated by a mixture of independent and constrained refinement
Δρ_max_, Δρ_min_ (e Å^−3^)	0.35, −0.24
Absolute structure	Flack *x* determined using 880 quotients [(*I* ^+^)−(*I* ^−^)]/[(*I* ^+^)+(*I* ^−^)] (Parsons *et al.*, 2013 ▸)
Absolute structure parameter	0.1 (4)

Computer programs: *SMART* and *SAINT* (Bruker, 2001 ▸), *SHELXT2014* (Sheldrick, 2015*a* ▸), *SHELXL2014* (Sheldrick, 2015*b* ▸), *Mercury* (Macrae *et al.*, 2008 ▸) and *PLATON* (Spek, 2009 ▸).

## supplementary crystallographic information

### Crystal data

**Table:**

C_5_H_7_N_2_^+^·C_8_H_5_O_4_^−^	*F*(000) = 272
*M**_r_* = 260.25	*D*_x_ = 1.444 Mg m^−^^3^
Monoclinic, *P*2_1_	Mo *K*α radiation, λ = 0.71073 Å
*a* = 5.1593 (6) Å	Cell parameters from 2425 reflections
*b* = 8.6124 (9) Å	θ = 2.2–24.7°
*c* = 13.5745 (19) Å	µ = 0.11 mm^−^^1^
β = 97.087 (4)°	*T* = 293 K
*V* = 598.56 (13) Å^3^	Block, colourless
*Z* = 2	0.26 × 0.24 × 0.20 mm

### Data collection

**Table:**

Bruker SMART APEX CCD area-detector diffractometer	*R*_int_ = 0.023
Radiation source: fine-focus sealed tube	θ_max_ = 25.0°, θ_min_ = 1.5°
ω scans	*h* = −6→6
6704 measured reflections	*k* = −10→10
2099 independent reflections	*l* = −16→14
1972 reflections with *I* > 2σ(*I*)	

### Refinement

**Table:**

Refinement on *F*^2^	Secondary atom site location: difference Fourier map
Least-squares matrix: full	Hydrogen site location: mixed
*R*[*F*^2^ > 2σ(*F*^2^)] = 0.059	H atoms treated by a mixture of independent and constrained refinement
*wR*(*F*^2^) = 0.186	*w* = 1/[σ^2^(*F*_o_^2^) + (0.0796*P*)^2^ + 0.5949*P*] where *P* = (*F*_o_^2^ + 2*F*_c_^2^)/3
*S* = 1.24	(Δ/σ)_max_ < 0.001
2099 reflections	Δρ_max_ = 0.35 e Å^−^^3^
180 parameters	Δρ_min_ = −0.24 e Å^−^^3^
1 restraint	Absolute structure: Flack *x* determined using 880 quotients [(*I*^+^)-(*I*^-^)]/[(*I*^+^)+(*I*^-^)] (Parsons *et al.*, 2013)
Primary atom site location: dual	Absolute structure parameter: 0.1 (4)

### Special details

**Table:**

Geometry. All esds (except the esd in the dihedral angle between two l.s. planes) are estimated using the full covariance matrix. The cell esds are taken into account individually in the estimation of esds in distances, angles and torsion angles; correlations between esds in cell parameters are only used when they are defined by crystal symmetry. An approximate (isotropic) treatment of cell esds is used for estimating esds involving l.s. planes.

### Fractional atomic coordinates and isotropic or equivalent isotropic displacement parameters (Å^2^)

**Table:**

	*x*	*y*	*z*	*U*_iso_*/*U*_eq_	
C11	0.5973 (12)	0.4659 (7)	0.8983 (5)	0.0401 (14)	
C12	0.7974 (14)	0.3719 (9)	0.9463 (6)	0.0545 (19)	
H12	0.8233	0.3657	1.0152	0.065*	
C13	0.9516 (13)	0.2908 (8)	0.8913 (7)	0.056 (2)	
H13	1.0809	0.2268	0.9232	0.067*	
C14	0.9221 (15)	0.3005 (8)	0.7882 (7)	0.057 (2)	
H14	1.0323	0.2466	0.7510	0.069*	
C15	0.7263 (13)	0.3915 (8)	0.7440 (5)	0.0489 (16)	
H15	0.7002	0.3992	0.6751	0.059*	
N11	0.5683 (10)	0.4714 (6)	0.7988 (4)	0.0370 (12)	
N12	0.4392 (12)	0.5509 (8)	0.9461 (4)	0.0532 (16)	
H12A	0.3216	0.6079	0.9134	0.064*	
H12B	0.4542	0.5492	1.0099	0.064*	
H1N	0.448 (13)	0.538 (8)	0.764 (5)	0.031 (15)*	
C21	0.1288 (10)	0.3445 (6)	0.3331 (4)	0.0302 (12)	
C22	0.0707 (13)	0.3464 (8)	0.4303 (4)	0.0421 (15)	
H22	−0.0615	0.2820	0.4472	0.050*	
C23	0.1998 (15)	0.4394 (9)	0.5026 (5)	0.0534 (18)	
H23	0.1531	0.4385	0.5666	0.064*	
C24	0.3968 (15)	0.5326 (9)	0.4797 (5)	0.0514 (17)	
H24	0.4850	0.5969	0.5278	0.062*	
C25	0.4648 (13)	0.5309 (8)	0.3840 (5)	0.0465 (15)	
H25	0.6035	0.5925	0.3697	0.056*	
C26	0.3347 (10)	0.4413 (7)	0.3089 (4)	0.0333 (13)	
C27	0.4332 (12)	0.4634 (9)	0.2093 (5)	0.0431 (15)	
C28	−0.0455 (11)	0.2378 (7)	0.2648 (4)	0.0335 (13)	
O21	0.6142 (12)	0.5515 (7)	0.2021 (4)	0.0696 (16)	
O22	0.3306 (11)	0.3890 (8)	0.1326 (4)	0.0627 (15)	
O23	−0.0154 (9)	0.2241 (6)	0.1742 (3)	0.0469 (11)	
O24	−0.2148 (10)	0.1635 (6)	0.3020 (3)	0.0488 (12)	
H22A	0.17 (2)	0.320 (11)	0.149 (7)	0.08 (3)*	

### Atomic displacement parameters (Å^2^)

**Table:**

	*U*^11^	*U*^22^	*U*^33^	*U*^12^	*U*^13^	*U*^23^
C11	0.039 (3)	0.039 (3)	0.043 (3)	−0.011 (3)	0.004 (3)	0.009 (3)
C12	0.052 (4)	0.052 (4)	0.055 (4)	−0.006 (3)	−0.014 (3)	0.020 (3)
C13	0.034 (3)	0.046 (4)	0.083 (6)	0.000 (3)	−0.008 (4)	0.016 (4)
C14	0.046 (4)	0.044 (4)	0.084 (6)	0.009 (3)	0.014 (4)	−0.003 (4)
C15	0.046 (3)	0.048 (4)	0.052 (4)	0.001 (3)	0.002 (3)	−0.007 (3)
N11	0.034 (2)	0.038 (3)	0.038 (3)	−0.002 (2)	0.002 (2)	0.006 (2)
N12	0.059 (4)	0.066 (4)	0.036 (3)	0.016 (3)	0.010 (3)	0.011 (3)
C21	0.029 (3)	0.031 (3)	0.030 (3)	0.004 (2)	0.003 (2)	0.001 (2)
C22	0.046 (3)	0.048 (4)	0.032 (3)	−0.006 (3)	0.006 (3)	−0.003 (3)
C23	0.068 (4)	0.058 (4)	0.033 (3)	0.000 (4)	0.001 (3)	−0.009 (3)
C24	0.056 (4)	0.053 (4)	0.042 (4)	−0.003 (3)	−0.006 (3)	−0.016 (3)
C25	0.039 (3)	0.047 (4)	0.051 (4)	−0.006 (3)	−0.001 (3)	−0.001 (3)
C26	0.031 (3)	0.032 (3)	0.036 (3)	0.004 (2)	0.003 (2)	0.002 (2)
C27	0.040 (3)	0.046 (3)	0.044 (4)	−0.001 (3)	0.007 (3)	0.011 (3)
C28	0.038 (3)	0.031 (3)	0.031 (3)	0.003 (3)	0.001 (2)	0.003 (2)
O21	0.067 (3)	0.073 (4)	0.075 (4)	−0.023 (3)	0.033 (3)	−0.001 (3)
O22	0.065 (3)	0.093 (4)	0.033 (2)	−0.016 (3)	0.015 (2)	0.001 (3)
O23	0.056 (3)	0.056 (3)	0.028 (2)	−0.009 (2)	0.0030 (19)	−0.006 (2)
O24	0.056 (3)	0.055 (3)	0.035 (2)	−0.021 (2)	0.002 (2)	−0.004 (2)

### Geometric parameters (Å, º)

**Table:**

C11—N12	1.325 (9)	C21—C28	1.519 (8)
C11—N11	1.341 (8)	C22—C23	1.374 (10)
C11—C12	1.407 (9)	C22—H22	0.9300
C12—C13	1.351 (11)	C23—C24	1.361 (11)
C12—H12	0.9300	C23—H23	0.9300
C13—C14	1.391 (12)	C24—C25	1.387 (10)
C13—H13	0.9300	C24—H24	0.9300
C14—C15	1.359 (10)	C25—C26	1.385 (9)
C14—H14	0.9300	C25—H25	0.9300
C15—N11	1.357 (9)	C26—C27	1.514 (9)
C15—H15	0.9300	C27—O21	1.216 (9)
N11—H1N	0.93 (7)	C27—O22	1.280 (9)
N12—H12A	0.8600	C28—O24	1.239 (7)
N12—H12B	0.8600	C28—O23	1.264 (7)
C21—C22	1.389 (8)	O22—H22A	1.08 (10)
C21—C26	1.421 (8)	O23—H22A	1.32 (11)
			
N12—C11—N11	118.4 (6)	C23—C22—C21	122.9 (6)
N12—C11—C12	123.5 (6)	C23—C22—H22	118.5
N11—C11—C12	118.1 (7)	C21—C22—H22	118.5
C13—C12—C11	119.3 (7)	C24—C23—C22	119.4 (7)
C13—C12—H12	120.3	C24—C23—H23	120.3
C11—C12—H12	120.3	C22—C23—H23	120.3
C12—C13—C14	121.8 (7)	C23—C24—C25	119.3 (6)
C12—C13—H13	119.1	C23—C24—H24	120.3
C14—C13—H13	119.1	C25—C24—H24	120.3
C15—C14—C13	117.5 (7)	C26—C25—C24	122.7 (7)
C15—C14—H14	121.2	C26—C25—H25	118.7
C13—C14—H14	121.2	C24—C25—H25	118.7
N11—C15—C14	120.9 (7)	C25—C26—C21	117.8 (6)
N11—C15—H15	119.5	C25—C26—C27	113.7 (6)
C14—C15—H15	119.5	C21—C26—C27	128.5 (5)
C11—N11—C15	122.3 (6)	O21—C27—O22	119.4 (6)
C11—N11—H1N	121 (4)	O21—C27—C26	119.7 (6)
C15—N11—H1N	117 (4)	O22—C27—C26	120.8 (6)
C11—N12—H12A	120.0	O24—C28—O23	121.7 (5)
C11—N12—H12B	120.0	O24—C28—C21	117.2 (5)
H12A—N12—H12B	120.0	O23—C28—C21	121.1 (5)
C22—C21—C26	117.9 (5)	C27—O22—H22A	112 (5)
C22—C21—C28	114.0 (5)	C28—O23—H22A	112 (4)
C26—C21—C28	128.1 (5)		
			
N12—C11—C12—C13	−179.0 (6)	C24—C25—C26—C27	177.2 (7)
N11—C11—C12—C13	−0.2 (9)	C22—C21—C26—C25	0.2 (8)
C11—C12—C13—C14	1.6 (11)	C28—C21—C26—C25	179.0 (6)
C12—C13—C14—C15	−1.9 (11)	C22—C21—C26—C27	−178.7 (6)
C13—C14—C15—N11	0.9 (10)	C28—C21—C26—C27	0.1 (9)
N12—C11—N11—C15	178.1 (6)	C25—C26—C27—O21	1.4 (9)
C12—C11—N11—C15	−0.8 (9)	C21—C26—C27—O21	−179.7 (6)
C14—C15—N11—C11	0.5 (10)	C25—C26—C27—O22	−179.5 (7)
C26—C21—C22—C23	1.2 (10)	C21—C26—C27—O22	−0.6 (10)
C28—C21—C22—C23	−177.7 (6)	C22—C21—C28—O24	−1.0 (7)
C21—C22—C23—C24	−1.1 (11)	C26—C21—C28—O24	−179.9 (6)
C22—C23—C24—C25	−0.6 (11)	C22—C21—C28—O23	−179.9 (6)
C23—C24—C25—C26	2.1 (11)	C26—C21—C28—O23	1.2 (8)
C24—C25—C26—C21	−1.9 (9)		

### Hydrogen-bond geometry (Å, º)

**Table:**

*D*—H···*A*	*D*—H	H···*A*	*D*···*A*	*D*—H···*A*
O22—H22*A*···O23	1.08 (10)	1.32 (10)	2.402 (7)	173 (9)
N11—H1*N*···O24^i^	0.93 (7)	1.78 (7)	2.705 (7)	180 (7)
N12—H12*A*···O23^i^	0.86	2.11	2.965 (7)	174
N12—H12*B*···O22^ii^	0.86	2.31	3.003 (8)	138
C12—H12···O23^iii^	0.93	2.53	3.374 (8)	151
C14—H14···O21^iv^	0.93	2.50	3.203 (9)	132

Symmetry codes: (i) −*x*, *y*+1/2, −*z*+1; (ii) *x*, *y*, *z*+1; (iii) *x*+1, *y*, *z*+1; (iv) −*x*+2, *y*−1/2, −*z*+1.
